# Stage-Specific *De Novo* Synthesis of Very-Long-Chain Dihydroceramides Confers Dormancy to *Entamoeba* Parasites

**DOI:** 10.1128/mSphere.00174-21

**Published:** 2021-03-17

**Authors:** Fumika Mi-ichi, Kazutaka Ikeda, Hiroshi Tsugawa, Sharmina Deloer, Hiroki Yoshida, Makoto Arita

**Affiliations:** a Division of Molecular and Cellular Immunoscience, Department of Biomolecular Sciences, Faculty of Medicine, Saga University, Saga, Japan; b Laboratory for Metabolomics, RIKEN Center for Integrative Medical Sciences, Yokohama, Japan; c Laboratory of Biomolecule Analysis, Department of Applied Genomics, Kazusa DNA Research Institute, Chiba, Japan; d Metabolome Informatics Research Team, RIKEN Center for Sustainable Resource Science, Yokohama, Japan; e Graduate School of Medical Life Science, Yokohama City University, Yokohama, Japan; f Division of Physiological Chemistry and Metabolism, Graduate School of Pharmaceutical Sciences, Keio University, Tokyo, Japan; University at Buffalo

**Keywords:** *Entamoeba*, amoebiasis, ceramide, encystation, infectious disease, lipidomics

## Abstract

Amoebiasis is a parasitic disease caused by Entamoeba histolytica infection and is a serious public health problem worldwide due to ill-prepared preventive measures as well as its high morbidity and mortality rates. Amoebiasis transmission is solely mediated by cysts. Cysts are produced by the differentiation of proliferative trophozoites in a process termed “encystation.” *Entamoeba* encystation is a fundamental cell differentiation process and proceeds with substantial changes in cell metabolites, components, and morphology, which occur sequentially in an orchestrated manner. Lipids are plausibly among these metabolites that function as key factors for encystation. However, a comprehensive lipid analysis has not been reported, and the involved lipid metabolic pathways remain largely unknown. Here, we exploited the state-of-the-art untargeted lipidomics and characterized 339 molecules of 17 lipid subclasses. Of these, dihydroceramide (Cer-NDS) was found to be among the most induced lipid species during encystation. Notably, in encysting cells, amounts of Cer-NDS containing very long *N*-acyl chains (≥26 carbon) were more than 30-fold induced as the terminal product of a *de novo* metabolic pathway. We also identified three ceramide synthase genes responsible for producing the very-long-chain Cer-NDS molecules. These genes were upregulated during encystation. Furthermore, these ceramide species were shown to be indispensable for generating membrane impermeability, a prerequisite for becoming dormant cyst that shows resistance to environmental assault inside and outside the host for transmission. Hence, the lipid subclass of Cer-NDS plays a crucial role for *Entamoeba* cell differentiation and morphogenesis by alternating the membrane properties.

**IMPORTANCE** *Entamoeba* is a protozoan parasite that thrives in its niche by alternating its two forms between a proliferative trophozoite and dormant cyst. Cysts are the only form able to transmit to a new host and are differentiated from trophozoites in a process termed “encystation.” During *Entamoeba* encystation, cell metabolites, components, and morphology drastically change, which occur sequentially in an orchestrated manner. Lipids are plausibly among these metabolites. However, the involved lipid species and their metabolic pathways remain largely unknown. Here, we identified dihydroceramides (Cer-NDSs) containing very long *N*-acyl chains (C_26_ to C_30_) as a key metabolite for *Entamoeba* encystation by our state-of-the-art untargeted lipidomics. We also showed that these Cer-NDSs are critical to generate the membrane impermeability, a prerequisite for this parasite to show dormancy as a cyst that repels substances and prevents water loss. Hence, ceramide metabolism is essential for *Entamoeba* to maintain the parasitic lifestyle.

## INTRODUCTION

**FIG 1:**
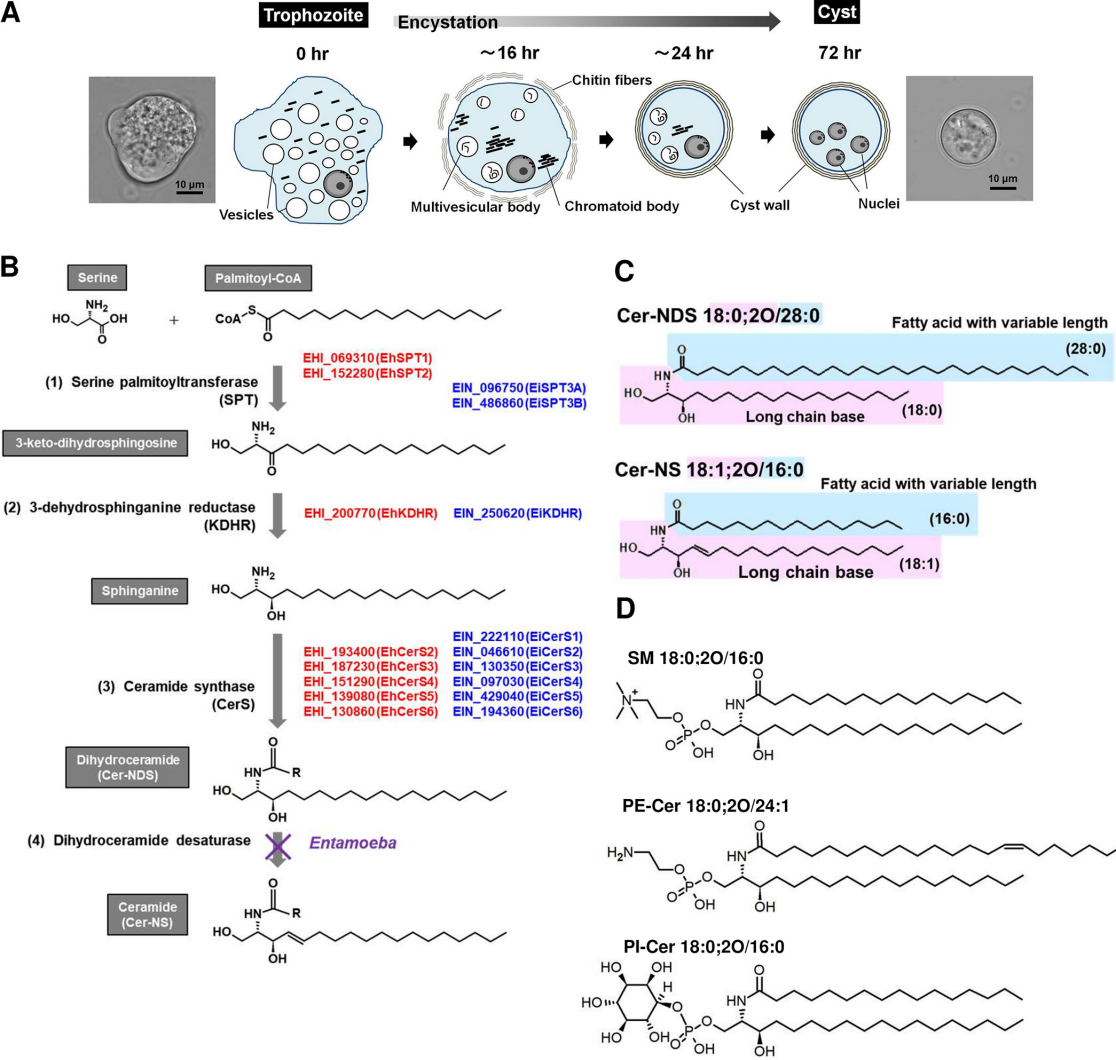
*Entamoeba* encystation and sphingolipid metabolism. (A) *Entamoeba* encystation. Schematic illustration of morphological and ultrastructural changes during encystation based on reference 11. Phase contrast microscopy images of trophozoite (0 h postinduction) and cyst (72 h postinduction) are shown. (B) *Entamoeba* atypical *de novo* ceramide synthesis pathway. AmoebaDB gene identifiers (IDs) for E. histolytica and *E. invadens* enzymes are indicated by red and blue colors, respectively. (C) Structures of Cer-NDS and Cer-NS. Cer 18:0;2O/28:0 and Cer 18:1;2O/16:0 are shown. (D) Structures of SM, PE-Cer, and PI-Cer. The most abundant species in *Entamoeba* cysts are shown.

Entamoeba histolytica, a protozoan parasite belonging to the clade Amoebozoa, causes amoebiasis, for which the development of new therapeutic means is urgently needed due to ill-prepared clinical options (1–3). As a parasitic strategy, E. histolytica alternates its form between a proliferative trophozoite and a dormant cyst (4, 5). The cyst is the only form able to transmit to a new host and is differentiated from trophozoites via stage transition, which is termed “encystation” (6). Encystation is a fundamental cell differentiation process, and the change in cell morphology is obvious; motile amoeboid cells become rounded nonmotile cells (Fig. 1A). Substantial changes also occur concurrently in cell components. For example, a single nucleus becomes four nuclei, and ribosomes become aggregated and form chromatoid bodies (7–11). Cells become coated with a cyst wall, and cell membrane permeability decreases greatly, resulting in mature cysts being rigid and resistant to environmental assault, such as desiccation (12–14). These structural and physiological changes are closely linked to fluctuations of various metabolites from diverse biochemical pathways, which play crucial roles in *Entamoeba* encystation (15). Chitins, a major component of the cyst wall, are specifically synthesized during encystation (16–18). Lipids, whose composition affects the physical properties of membranes, such as fluidity and rigidity (19), are plausibly responsible for the decrease in membrane permeability of the *Entamoeba* cyst. However, the lipid species involved have not been identified, and their metabolic pathways remain largely unknown. In this study, to identify the lipid species fluctuating during *Entamoeba* encystation, we performed state-of-the-art liquid chromatography-mass spectrometry (LC-MS)-based untargeted lipidomics (20) and found ceramides containing nonhydroxy fatty acid and dihydrosphingosine (Cer-NDSs) to be among the most induced lipid species.

Ceramides play versatile roles in homeostasis (21, 22). They are pivotal intermediates in the synthesis of a variety of sphingolipids that are essential membrane components, such as sphingomyelin (SM) and ganglioside. Ceramides and their derivatives also function as signaling molecules in cell proliferation, differentiation, and death. Typically, the ceramides are produced by *de novo* synthesis and salvage pathways. The *de novo* pathway consists of four sequential biochemical reactions (Fig. 1B) (21, 22): (i) condensation of serine and palmitoyl-coenzyme A (CoA), the rate-limiting step; (ii) reduction of the resulting 3-keto-dihydrosphingosine; (iii) acylation of hydroxyl species using acyl-CoA; and (iv) desaturation of the dihydro product, Cer-NDS. The salvage pathway includes SM hydrolysis by phospholipase C and sphingolipid degradation and recycling to provide intermediates for the *de novo* pathway (19, 23, 24). In *Entamoeba*, the presence of sphingolipids and the importance of sphingolipid metabolism in trophozoite proliferation, encystation, and excystation were previously described (25–30). Of note, based on AmoebaDB (http://amoebadb.org/amoeba/), *Entamoeba* possesses an atypical *de novo* pathway for ceramide synthesis in contrast to typical free-living organisms, such as humans and yeast; the gene encoding the fourth enzyme in the *de novo* pathway, dihydroceramide desaturase, is absent from the *Entamoeba* genome (see Fig. 1B). In this study, we conducted comprehensive nontargeted lipidomics and successfully identified Cer-NDS containing very long *N*-acyl chains (C_26_ to C_30_) (see Fig. 1C for the structure) as one of the most induced lipid species during *Entamoeba* encystation.

## RESULTS

### Identification of lipid species and their fluctuating levels (increase or decrease) during encystation.

**FIG 2:**
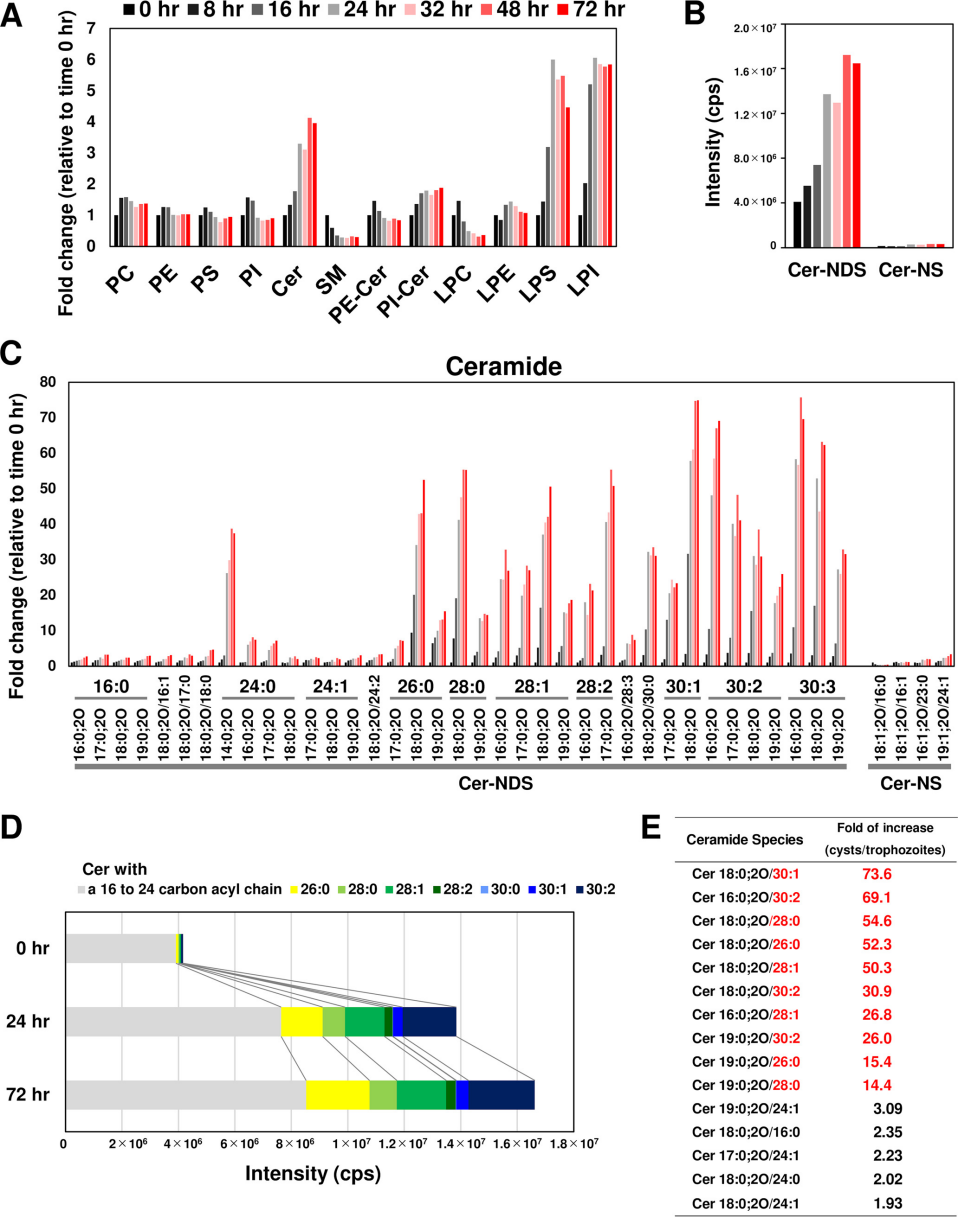
Comprehensive analysis of lipid species during *Entamoeba* encystation by untargeted lipidomics. (A) Fluctuation of major lipid classes during encystation. Signal intensity levels are shown as fold change relative to the level at time zero. Time course profiles of each lipid species are presented in Fig. S1 in the supplemental material. (B) Comparison of the total intensity of Cer-NS and Cer-NDS detected in encysting cells at the indicated times. The colors used for indicating the time are as in panel A. (C) Changes in the ceramide species profile during *Entamoeba* encystation. LC-MS/MS signal intensity levels are shown as fold change relative to the level at time zero. The colors used for indicating the time are as in panel A. (D) Dynamics of the increased levels of a broad range of ceramides. Stacked bar graph of ceramide species, which were detected in encysting cells at 0, 24, and 72 h after encystation induction and classified based on their acyl chains, are shown with different colors. (E) List of 15 most abundant ceramide species in cysts (72 h after induction), all of which were reproducibly detected in three independent experiments (Table S1). Red letters indicate ceramide species whose levels were >10-fold higher than those in trophozoites (0 h after induction). Representative data (Sample 1 in Table S1) are shown from three independent experiments.

To comprehensively investigate the lipid species that fluctuate during encystation, we used *in vitro* culture of Entamoeba invadens. Generally, studies of *Entamoeba* encystation have adopted the *in vitro* culture of *E. invadens*, a reptilian parasite, and not that of E. histolytica as a model system (see Fig. 1A). This is because the strains of E. histolytica available in the laboratory do not encyst after adaptation to culture conditions. The *E. invadens* life cycle is the same as that of E. histolytica, and the symptoms caused by *E. invadens* infection are similar to those of E. histolytica (4, 5). Lipids were extracted from encysting *E*. *invadens* cells at designated time points after the induction of encystation and then were analyzed by untargeted lipidomics. During encystation, a series of lipid species, including ceramide, ceramide phosphatidylinositol (PI-Cer) (see Fig. 1D for the structure), lysophosphatidylserine (LPS), and lysophosphatidylinositol (LPI) was significantly increased with time (Fig. 2A and see Fig. S1 in the supplemental material; see Fig. 1A for the morphological and ultrastructural changes). In contrast, levels of SM (see Fig. 1D for the structure) and lysophosphatidylcholine (LPC) species were significantly decreased. No significant fluctuations in phospholipid or other sphingolipid species (phosphatidylcholine [PC], phosphatidylethanolamine [PE], PI, phosphatidylserine [PS], and ceramide phosphoethanolamine [PE-Cer]) (see Fig. 1D for the structure) were observed throughout encystation. Ceramide molecules were detected throughout the *Entamoeba* life cycle (trophozoite and cyst stages) and mainly comprised Cer-NDSs (Fig. 2B). In mammals, the ceramide lipid class is essential to prevent water loss from the skin (31). *Entamoeba* cysts are also resistant to desiccation (5, 32). We, therefore, focused on Cer-NDS species for further analyses.

10.1128/mSphere.00174-21.1 FIG S1 Changes of ceramide and major lipid species profiles during *Entamoeba* encystation. LC-MS/MS signal intensity levels of ceramide (A), PE-Cer (B), PI-Cer (C), SM (D), PC (E), PE (F), PS (G), PI (H), LPC (I), LPE (J), LPS, and LPI (K) are shown. Representative data are shown from three independent experiments. Download FIG S1, TIF file, 1.6 MB. Copyright © 2021 Mi-ichi et al. 2021 Mi-ichi et al. https://creativecommons.org/licenses/by/4.0/ This content is distributed under the terms of the Creative Commons Attribution 4.0 International license.

10.1128/mSphere.00174-21.8 TABLE S1 Ceramide species reproducibly detected as the 15 most abundant species in three independent experiments. Download Table S1, PDF file, 0.01 MB. Copyright © 2021 Mi-ichi et al. 2021 Mi-ichi et al. https://creativecommons.org/licenses/by/4.0/ This content is distributed under the terms of the Creative Commons Attribution 4.0 International license.

In *Entamoeba* trophozoites (*E*. *invadens* cells before encystation induction), Cer 18:0;2O/24:1, Cer 18:0;2O/24:0, Cer 19:0;2O/24:1, Cer 18:0;2O/16:0, and Cer 17:0;2O/24:1 were dominantly present (0 h in Fig. S1A), and the amount of these species increased by ≤3-fold during the course of encystation (Fig. 2C and E and Fig. S1A). In contrast, the amounts of very-long-chain Cer-NDS species, such as Cer 18:0;2O/30:1, Cer 16:0;2O/30:2, and Cer 18:0;2O/28:1, were increased 10- to 80-fold between 16 and 24 h after encystation induction (Fig. 2C and E). At 72 h, the abundance of very-long-chain Cer-NDS species became evident (Fig. 2D). Among those ceramides consistently detected in three independent experiments (see Table S1), 10 species of very-long-chain Cer-NDS (≥26 acyl chain) were significantly elevated (Fig. 2E and Table S1).

### Revealing a *de novo* ceramide synthesis pathway in *Entamoeba*.

Very-long-chain Cer-NDSs were not detected in bovine serum, which is the major lipid source in *Entamoeba* encystation-inducing culture medium (33); therefore, it was unlikely that very-long-chain Cer-NDSs were derived from the external milieu. Of interest, all necessary genes for the *de novo* ceramide synthesis are harbored by both the E. histolytica and *E. invadens* genomes except for one gene encoding dihydroceramide desaturase (Fig. 1B) (AmoebaDB, http://amoebadb.org/amoeba/); there are two types of genes encoding serine palmitoyl transferase (SPT), one gene for 3-dehydrosphinganine reductase (KDHR), and five (E. histolytica) or six (*E. invadens*) genes for ceramide synthase (CerS) (27).

**FIG 3:**
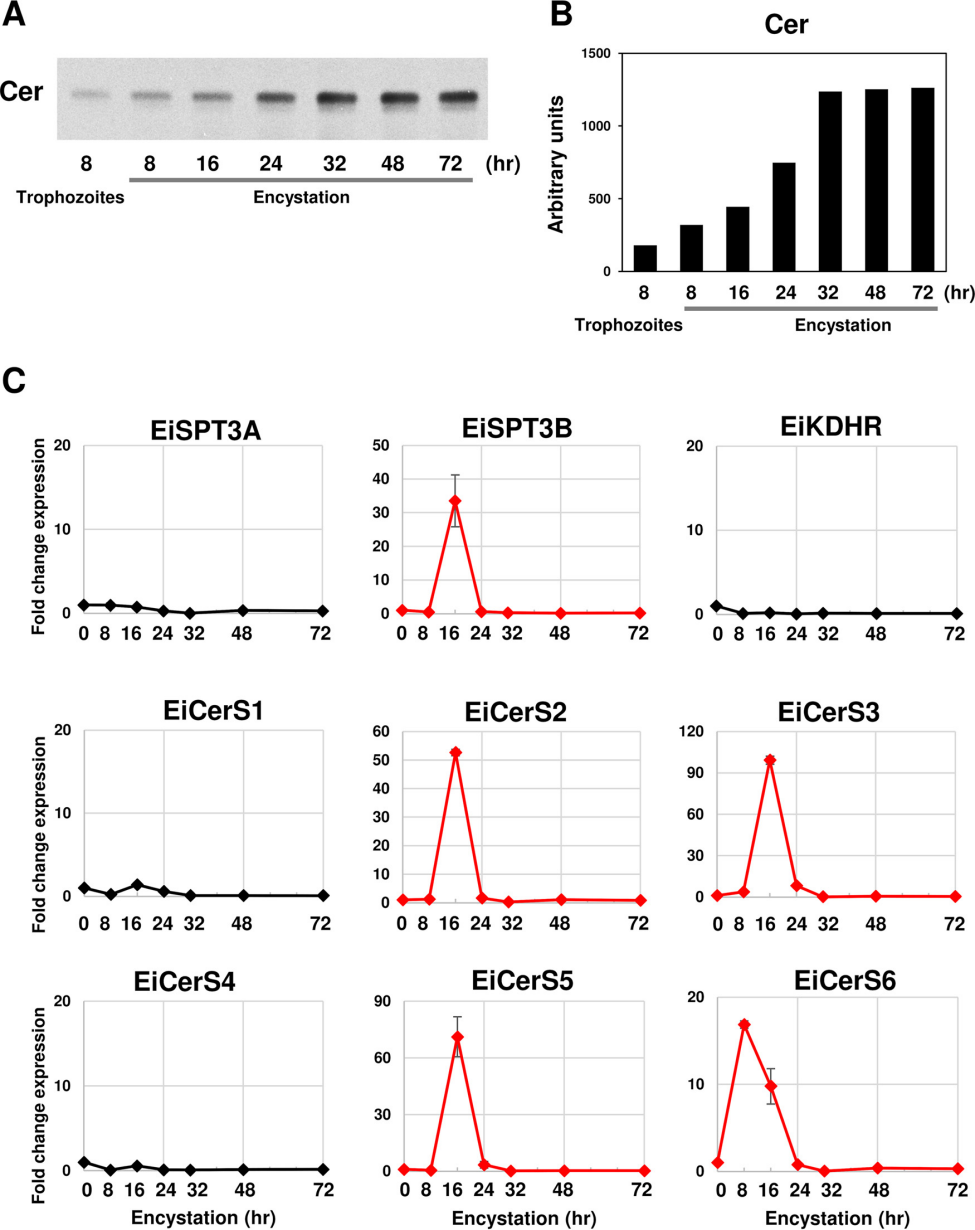
*De novo* ceramide synthesis is elevated during *Entamoeba* encystation. (A) Time course of ceramide accumulation in *E. invadens* encysting cells. TLC of lipids extracted from encysting cells, which were metabolically labeled with [^14^C]serine. (B) Quantification of the ceramide bands in panel A by densitometric analysis. (C) Transcriptional changes of the genes encoding the enzymes involved in *de novo* ceramide synthesis during encystation. Expression levels are shown as fold changes at the indicated time points after the induction of encystation relative to the level at time zero. Experiments were performed in triplicates, and representative data are shown from three independent experiments.

To show the capability of *Entamoeba* to synthesize ceramides *de novo*, proliferating trophozoites and encysting cells were metabolically labeled with l-[U-^14^C]serine, a substrate for the first enzyme (SPT) in the *de novo* pathway (see Fig. 1B). ^14^C-labeled bands corresponding to ceramides were detected in both trophozoites and encysting cells (Fig. 3A). During encystation, an accumulation of radiolabeled ceramide with time was observed. A dramatic increase of radiolabeled ceramide was observed between 16 and 32 h (Fig. 3B). Alkaline treatment did not change the intensity of the detected bands, ruling out the lipids being glycerolipids (see Fig. S2). These results clearly indicated that *Entamoeba* synthesized ceramides by *de novo* biosynthesis. Notably, the time course for the accumulation of ^14^C-labeled ceramide correlated well with the increased amount of very-long-chain Cer-NDSs between 16 and 24 h after encystation induction and reached a plateau after 24 h (Fig. 2C and Fig. S1A). Consistently, during the initiation phase of encystation, expression of a series of ceramide biosynthetic enzymes was coordinately induced in *Entamoeba* (Fig. 3C). These results indicated that the induction of very-long-chain Cer-NDSs during *Entamoeba* encystation appeared to be mediated by *de novo* biosynthesis.

10.1128/mSphere.00174-21.2 FIG S2 Alkaline hydrolysis of l-[U-^14^C]serine-labeled lipids. Lipids extracted from *Entamoeba* cells metabolically labeled with [^14^C]serine for 72 h in the encystation induction culture (Fig. 3A) (72 h) were either alkaline hydrolyzed (+) or not (−). Download FIG S2, TIF file, 0.9 MB. Copyright © 2021 Mi-ichi et al. 2021 Mi-ichi et al. https://creativecommons.org/licenses/by/4.0/ This content is distributed under the terms of the Creative Commons Attribution 4.0 International license.

### Identification of the ceramide synthase gene responsible for producing Cer-NDSs in *Entamoeba*.

**FIG 4:**
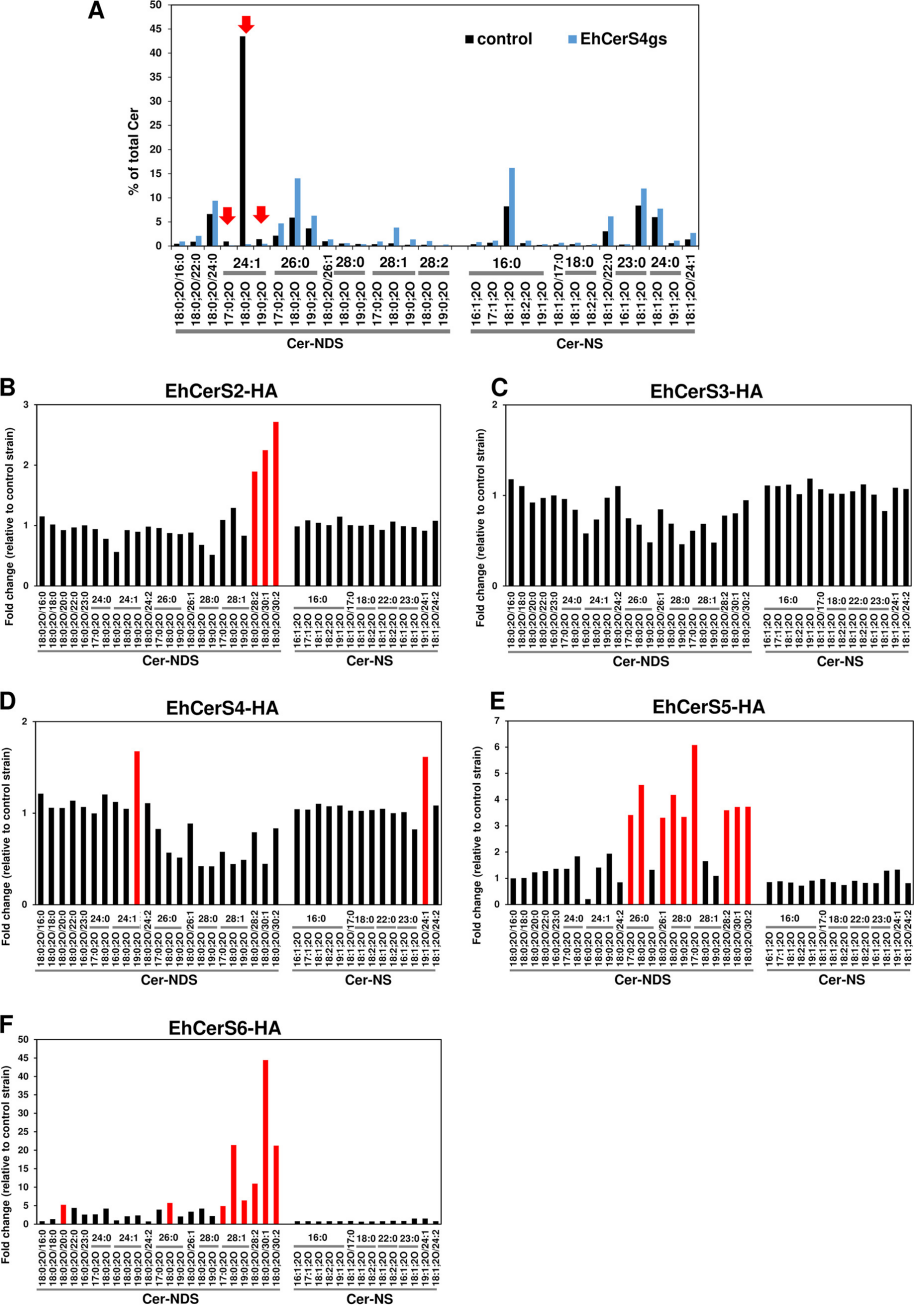
Knockdown (A) and overexpression (B to F) of CerS genes change the ceramide profile in E. histolytica. (A) Percentages of each ceramide species relative to the total amount in EhCerS4gs and the control strain. Red arrows indicate the ceramide species showing the most significant differences. Changes in the levels of ceramide species in EhCerS2-HA (B), EhCerS3-HA (C), EhCerS4-HA (D), EhCerS5-HA (E), and EhCerS6-HA (F) strains. Signal intensity levels are shown as fold change to that of the control strain. Red bars indicate the ceramide species increased by >1.5-fold (B to D), 3-fold (E), and 5-fold (F). Representative data are shown from two independent experiments.

Variation in the acyl chain length of Cer-NDSs observed during *Entamoeba* encystation is likely to be generated by different CerS isozymes, as observed in other organisms (21, 22). To identify the CerS responsible for very-long-chain Cer-NDS biosynthesis in *Entamoeba*, we exploited an approach combining genetics and lipidomics. The genetic approach included gene knockdown mediated by transcriptional gene silencing via antisense small RNA (34, 35) and gene overexpression (36). We used E. histolytica instead of *E*. *invadens* as the host because the genetic systems for *E*. *invadens* have not been widely adopted. In E. histolytica trophozoites, Cer-NDS species were similarly detected as in *E. invadens* trophozoites (see Fig. S3A). A gene knockdown experiment was performed using five E. histolytica gene silencing (gs) transformants, EhCerS2gs to EhCerS6gs, in each of which a single gene among the five *EhCerS*s was knocked down. Note that E. histolytica does not have a counterpart of *E. invadens CerS1* (*EiCerS1*) (see Fig. 1B). After verifying the level of gene knockdown in each transformant by quantitative reverse transcription-PCR (qRT-PCR) (Fig. S3B), the lipidomic profiles of Cer-NDS species in all EhCerSgs (except for EhCerS3gs) and mock transformants were individually determined (Fig. 4A and Fig. S3C to E). One transformant, EhCerS3gs, showed a severe growth defect, which hampered long-term subculture. Among the transformants tested, only EhCerS4gs showed a significant reduction in Cer-NDS levels; the most significant reduction was observed in Cer 18:0;2O/24:1, and the amounts of Cer 17:0;2O/24:1 and Cer 19:0;2O/24:1 were also reduced (Fig. 4A). In EhCerS4gs, both *EhCerS4* and *EhCerS5* transcripts were significantly downregulated (0.25% ± 0.03% and 4.2% ± 0.3%, respectively, relative to the mock transformant 100% control) (Fig. S3B). However, a contribution of EhCerS5 was ruled out because the amounts of Cer-NDS species were not changed in EhCerS5gs, in which only the *EhCerS5* transcript was reduced (4.4% ± 0.6%) (Fig. S3B and D). These results indicated that EhCerS4 was responsible for synthesizing Cer-NDSs containing a C_24:1_ acyl chain. None of the remaining three transformants (EhCer2gs, -5gs, and -6gs) showed obvious changes in their Cer-NDS species profile, probably because of genetic redundancy (Fig. S3C to E).

10.1128/mSphere.00174-21.3 FIG S3 Gene knockdown of CerS2 to -6 in E. histolytica. (A) Comparison of the total intensity of Cer-NS and Cer-NDS detected in E. histolytica trophozoites. Experiments were performed in triplicates; *n* = 3. (B) Reduced levels of CerS2 to -6 transcripts in gene knockdown strains. qRT-PCR data of CerS2 to -6 transcripts in the gene knockdown and the control strains are shown. The level of each CerS transcript is expressed as a percentage in the gene knockdown strain relative to that in the control strain. Experiments were performed in triplicates; *n* = 3. Abundance of ceramide species in each CerS gene knockdown [EhCerS2gs (C), EhCerS5gs (D), and EhCerS6gs (E)] and control strain. Representative data are shown from two independent experiments. Download FIG S3, TIF file, 1.7 MB. Copyright © 2021 Mi-ichi et al. 2021 Mi-ichi et al. https://creativecommons.org/licenses/by/4.0/ This content is distributed under the terms of the Creative Commons Attribution 4.0 International license.

Overexpression experiment of each *EhCerS*s was also performed; each *EhCerS* gene (see Fig. 1B) was separately overexpressed as a hemagglutinin (HA)-tagged protein to yield E. histolytica transformants, namely, EhCerS2-HA to EhCerS6-HA (Fig. 4B to F). In EhCerS2-HA, EhCerS5-HA, and EhCerS6-HA, only the targeted *EhCerS* was selectively upregulated (see Fig. S4A). In EhCerS2-HA, levels of Cer 18:0;2O/28:2, Cer 18:0;2O/30:1, and Cer 18:0;2O/30:2 were selectively increased (Fig. 4B). In EhCerS5-HA, levels of Cer 17:0;2O/26:0, Cer 18:0;2O/26:0, Cer 18:0;2O/26:1, Cer 18:0;2O/28:0, Cer 19:0;2O/28:0, Cer 17:0;2O/28:1, Cer 18:0;2O/28:2, Cer 18:0;2O/30:1, and Cer 18:0;2O/30:2 were selectively increased (Fig. 4E). In EhCerS6-HA, levels of Cer 18:0;2O/20:0, Cer 18:0;2O/26:0, Cer 17:0;2O/28:1, Cer 18:0;2O/28:1, Cer 19:0;2O/28:1, Cer 18:0;2O/28:2, Cer 18:0;2O/30:1, and Cer 18:0;2O/30:2 were selectively increased (Fig. 4F). These results indicate that variation of acyl chain length in Cer-NDSs was generated by ectopic overexpression of CerS isozymes. EhCerS2 produces C_28:2_-, C_30:1_-, and C_30:2_-Cer-NDSs, EhCerS5 produces C_26:0_-, C_26:1_-, C_28:0_-, C_28:1_-, C_28:2_-, C_30:1_-, and C_30:2_-Cer-NDSs, and EhCerS6 produces C_20:0_-, C_26:0_-, C_28:1_-, C_28:2_-, C_30:1_-, and C_30:2_-Cer-NDSs. These results were consistent with the encysting *E*. *invadens* cells; the transcription levels of *EiCerS2*, -*5*, and -*6*, were significantly upregulated (Fig. 3C), while the amount of Cer-NDS species containing C_26:0_, C_28:0_, C_28:1_, C_28:2_, C_30:1_, and C_30:2_ were substantially increased (Fig. 2C). Overlap in the Cer-NDSs produced by EhCerS2, -5, and -6 reinforces our premise that genetic redundancy among these three CerSs results in these single gene knockdown strains having no mutant phenotype. Of note, EhCerS6-HA, in which Cer-NDS levels were dramatically increased (Fig. 4F), displayed a growth defect (Fig. S4B). This may have resulted from the toxicity of a very high level of Cer-NDSs that accumulated in trophozoites. EhCerS4-HA showed significant increase of Cer 19:0;2O/24:1 and Cer 19:1;2O/24:1 compared to that in the control (Fig. 4D). Therefore, EhCerS4 appeared to be responsible for synthesizing Cer-NDS with a C_24:1_ acyl chain, which does not overlap the Cer-NDS species synthesized by functionally redundant EhCerS2, -5, and -6. EhCerS3-HA did not show obvious changes in Cer-NDS levels (Fig. 4C). These results indicated that the variation of Cer-NDS species in *Entamoeba* was generated by the different CerS isozymes (Table 1).

**TABLE 1:** Variety of dihydroceramide species generated in Entamoeba histolytica by ceramide synthase isozymes (EhCerS2 to -6)

Product	EhCerS2	EhCerS3	EhCerS4	EhCerS5	EhCerS6
Dihydroceramide species		nd^*a*^			
					C_20:0_-Cer
			C_24:1_-Cer		
				C_26:0_-Cer	C_26:0_-Cer
				C_26:1_-Cer	
				C_28:0_-Cer	
				C_28:1_-Cer	C_28:1_-Cer
	C_28:2_-Cer			C_28:2_-Cer	C_28:2_-Cer
	C_30:1_-Cer			C_30:1_-Cer	C_30:1_-Cer
	C_30:2_-Cer			C_30:2_-Cer	C_30:2_-Cer

a nd, not determined.

10.1128/mSphere.00174-21.4 FIG S4 Overexpression of *CerS2* to *CerS6* in E. histolytica. (A) Levels of *CerS2* to *CerS6* overexpression in EhCerS2-HA to EhCerS6-HA strains. qRT-PCR data of *CerS2* to *CerS6* transcripts in the overexpression and control strains are shown. The level of each *CerS* transcript is expressed as fold change relative to that of the control strain. Experiments were performed in triplicates; *n* = 3. (B) Growth curves of EhCerS2-HA to EhCerS6-HA and control strains. Experiments were performed in triplicates; *n* = 3. Download FIG S4, TIF file, 1.5 MB. Copyright © 2021 Mi-ichi et al. 2021 Mi-ichi et al. https://creativecommons.org/licenses/by/4.0/ This content is distributed under the terms of the Creative Commons Attribution 4.0 International license.

### Ceramide metabolism in *Entamoeba*.

**FIG 5:**
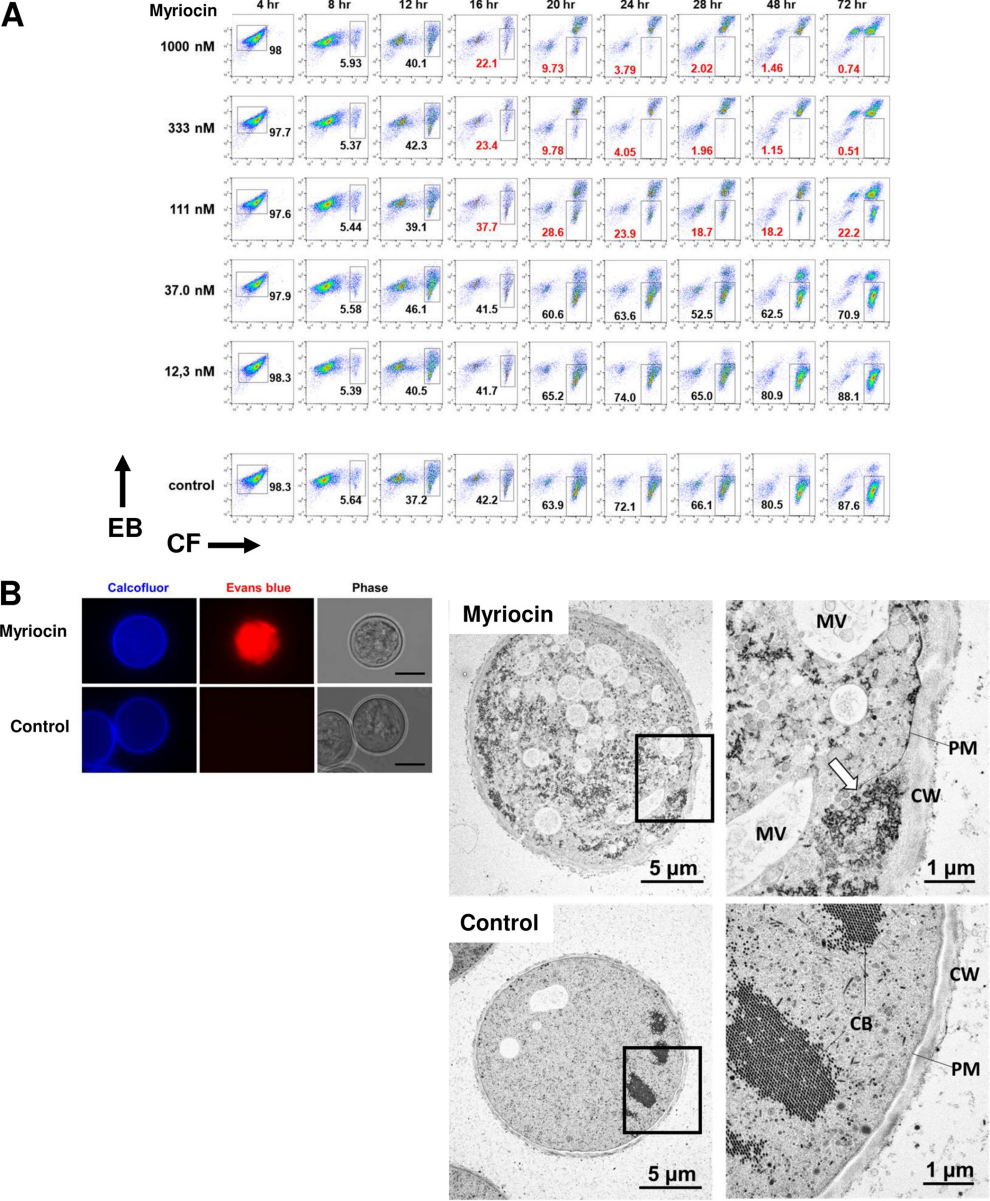
Effects of myriocin on *Entamoeba* cyst formation. (A) Encystation assay. Immediately after induction, *E. invadens* was cultivated in encystation medium in the presence of various concentrations of myriocin (111 to 1,000 nM). Flow cytometry results obtained at the indicated times after induction are shown. The number inside each panel indicates the percentage of the boxed cell population. The red numbered panels indicate the conditions under which myriocin had an effect on encysting cells. EB, Evans blue; CF, calcofluor. (B, left) Fluorescence microscopy images of a cell treated with 1 μM myriocin or a control cell observed at 24 h after encystation induction. Bars, 10 μm. Representative images are shown from two independent experiments. (Right) Electron microscopy images of 1 μM myriocin-treated (top) or control (bottom) cells observed at 24 h after encystation induction (left, whole cells; right, magnified images of boxed areas). Arrow indicates the disconnected plasma membrane. Representative images from more than 20 sections are shown. CB, chromatoid body; CW, cyst wall; MV, multivesicular body; PM, plasma membrane.

To understand ceramide metabolism in *Entamoeba*, we investigated the effect of myriocin, a known inhibitor for the first enzyme (SPT) in the *de novo* pathway for ceramide biosynthesis (see Fig. 1B). Myriocin dose-dependently inhibited cyst formation in *in vitro* cultures of *E. invadens*, which was consistent with the previous report (27, 28). The 50% inhibitory concentration [IC_50_] was calculated as 68.6 ± 12.5 nM (*n* = 3) (Fig. 5A). Also, the physiological changes during the course of encystation were monitored by flow cytometry (37). Evans blue (EB) was used as an indicator of membrane permeability, and calcofluor (CF) was used as an indicator of the level of chitin, a major component of the cyst wall (38). As shown in the control in Fig. 5A, it appeared that the CF^−^ EB^+^ population (proliferating trophozoites) was gradually changed to a CF^+^ EB^−^ population (mature cysts) through CF^low^ EB^+^ and CF^+^ EB^+^ populations. At 12 h after induction, myriocin treatment did not affect the phenotype, but at 16 h, cell differentiation was paused, resulting in the accumulation of an irregular CF^+^ EB^strong^ population (abnormal cells) at 20 h. These results indicated that myriocin impaired the encystation process at 16 to 20 h postinduction. Importantly, this time frame correlated well with the lipidomic changes of very-long-chain Cer-NDSs dramatically increased between 16 and 24 h after induction of encystation (Fig. 2C). These results indicated that inhibition of very-long-chain Cer-NDS biosynthesis by myriocin halted cyst formation.

Next, we determined the consequence of *Entamoeba* encysting cells treated with myriocin. After 24 h, when the effect of myriocin was first apparent on cyst formation, both control and myriocin-treated live cells were stained by CF and EB (Fig. 5B). Flow cytometry analysis showed that the level of CF fluorescence in myriocin-treated cells was comparable to that of untreated cells (Fig. 5A). A change in the CF signal reflects the synthesis and degradation of chitin polymers. Therefore, these results indicate that chitins are synthesized and placed in the cyst wall at similar levels in both myriocin-treated and untreated cells. However, a distinct physiological change was observed in myriocin-treated cells. The fluorescence signal of EB, an indicator of membrane permeability, abnormally accumulated inside the cells, indicating that myriocin treatment increased the membrane permeability of encysting cells (Fig. 5B).

To further determine the structural changes induced by myriocin treatment, we performed transmission electron microscopy analysis of encysting cells in either the presence or absence of myriocin (Fig. 5C). Cells were prepared by rapid freezing and freeze-substitution to preserve the membrane structure (39). The myriocin-treated cells were withered, and accumulations of abnormal vacuoles were observed throughout the cytoplasm. Notably, the cell membranes of myriocin-treated cells were more compressed and disconnected. Furthermore, the cyst wall regions of treated cells were swollen, and cell components randomly filled the spaces between the regions of disrupted membranes and the cyst walls. It is worth mentioning that obvious changes in lipidome of encysting cells (24 h postinduction) by myriocin treatment was observed in Cer-NDSs containing very long *N*-acyl chains (≥26 carbon) and their metabolites, PI-Cers. The level of LPS was also affected by myriocin treatment, but to a small extent compared to that for Cer-NDSs (see Fig. S5; Table S2). These results indicate that the off-target effect of myriocin on lipid metabolism in *Entamoeba* encysting cells was quite limited under this experimental condition. These results indicated that the stage-specific induction of Cer-NDSs with very long *N*-acyl chains (C_26_ to C_30_) were indispensable to develop membrane impermeability.

10.1128/mSphere.00174-21.5 FIG S5 Comparison of lipid profiles for myriocin-treated and untreated *E. invadens* encysting cells. Time course profiles (16 to 24 h postinduction) of each lipid species determined by untargeted lipidomics are shown as fold change of signal intensity levels relative to those at time 0 h. The 10 most abundant species of each lipid class except for LPS and LPI are summarized in the top graphs, whereas the 4 most abundant species of LPS and LPI are summarized in the bottom graph. Representative data are shown from three independent experiments (Table S2). Download FIG S5, TIF file, 1.3 MB. Copyright © 2021 Mi-ichi et al. 2021 Mi-ichi et al. https://creativecommons.org/licenses/by/4.0/ This content is distributed under the terms of the Creative Commons Attribution 4.0 International license.

10.1128/mSphere.00174-21.9 TABLE S2 Lipid metabolome for Fig. S5. Lipid profiles were detailed with the mass spectrometry properties, including precursor *m/z*, retention time, adduct type, signal-to-noise ratio, isotopic pattern, and MS/MS spectrum for each lipid ion. The metabolite name was determined by the nomenclature system suggested in lipidomics standards initiative (see reference 20 for details). The ion peak height was used as the quantitative value for lipid molecules. Download Table S2, XLSX file, 0.2 MB. Copyright © 2021 Mi-ichi et al. 2021 Mi-ichi et al. https://creativecommons.org/licenses/by/4.0/ This content is distributed under the terms of the Creative Commons Attribution 4.0 International license.

## DISCUSSION

*Entamoeba* encystation is a crucial process for maintaining the life cycle of this parasitic species. Encystation is a fundamental cell differentiation and morphogenesis process that involves a variety of pathways, which function as an orchestrated network (5). Here, we performed an untargeted lipidomic analysis of encysting cells over time. This approach enabled us to reveal that the levels of Cer-NDSs were significantly induced during encystation. We also unraveled unique features of an *Entamoeba* metabolic pathway and its physiology. First, *Entamoeba* possesses an atypical *de novo* ceramide synthesis pathway that produces Cer-NDSs possessing a broad range of acyl chains (C_16_, C_20_, C_22_, C_23_, C_24_, C_26_, C_28_, C_30:0_, C_24:1_, C_28:1_, C_28:2_, C_28:3_, C_30:1_, and C_30:2_) as terminal metabolites. Second, during encystation, the amounts of very-long-chain Cer-NDSs with an acyl chain length of ≥26 were increased, coinciding with transcriptional upregulation of the three genes encoding CerS2, -5, and -6. Those enzymes were functionally redundant and responsible for producing those ceramides. Third, inhibition of *de novo* ceramide synthesis caused damage to the plasma membrane and increased membrane permeability to a nonphysiological level, resulting in the formation of aberrant cysts.

Differentiation into dormant cysts from proliferative trophozoites is necessary for *Entamoeba* to be resistant to environmental assaults inside as well as outside the host and to be transmitted to a new host. This dormant process involves rounding and strengthening of the cells, which requires alternations to properties of the plasma membrane, such as changes to the composition and topology of the lipid bilayer. Halting Cer-NDS production by adding myriocin to *in vitro* encystation induction cultures resulted in proliferating trophozoites becoming rounded as normal but that differentiated into aberrant cysts. Therefore, newly synthesized Cer-NDSs in encysting cells are not involved in the cell rounding process, but they do have crucial roles after the encysting cells became rounded. The underlying molecular mechanisms need to be elucidated; however, in myriocin-treated encysting cells, a partly disconnected plasma membrane and abnormal accumulation in the cytoplasm of EB, a membrane impermeable dye, were simultaneously observed. Furthermore, we can estimate the content of very-long-chain Cer-NDSs with the acyl chain length of ≥26 in a single encysting cell to be significantly elevated because cell numbers should not theoretically increase, as encystation is a differentiation process, but they empirically decreased a bit. Cell volume also became around half during encystation. These findings indicate that very-long-chain Cer-NDSs have critical roles in maintaining the plasma membrane impermeability and that *Entamoeba* regulates intracellular ceramide metabolism to provide very-long-chain Cer-NDSs to enable transmission to a new host. Cer-NSs were also present in *Entamoeba* despite the absence of dihydroceramide desaturase gene in *Entamoeba* genome (AmoebaDB). Therefore, these ceramides containing nonhydroxy fatty acid and sphingosines (Cer-NSs) are plausibly acquired from the culture medium. Interestingly, a species difference of *Entamoeba* in the ratio of Cer-NS to ceramide was seen (Fig. 2B and see Fig. S3A in the supplemental material). This may reflect that the E. histolytica capability for *de novo* ceramide synthesis is less than that of *E. invadens*.

Furthermore, we showed evidence that Cer-NDS species may also be essential for *Entamoeba* to multiply as trophozoites. Both E. histolytica and *E. invadens* trophozoite proliferations were impaired by myriocin with IC_50_ values of 46. 7 ± 11.5 nM (*n* = 3) and 1.90 ± 0.10 μM (*n* = 3), respectively. The growth impairment by myriocin was complemented by the gene knockdown analyses targeting the enzymes in the E. histolytica *de novo* ceramide biosynthesis pathway (see Fig. 1B). Both knockdown strains, EhSPT1gs and EhSPT2gs (see Fig. S6A), showed severe growth defects, which hampered long-term subculture. Another knockdown strain, EhKDHRgs, also showed a growth defect (Fig. S6A and B). Another knockdown strain, EhCerS3gs, in which the downregulation of *EhCerS3* was confirmed in an early subculture (Fig. S3B), showed severe growth defects, similar to those of EhSPT1gs and EhSPT2gs. Alternating its forms between a proliferating trophozoite and dormant cyst is a parasitic strategy for surviving in different niches. Hence, ceramide metabolism plays crucial roles in the *Entamoeba* life cycle.

10.1128/mSphere.00174-21.6 FIG S6 Gene knockdown of the first and second enzymes of the *Entamoeba de novo* ceramide synthesis pathway. (A) Reduced levels of target gene transcripts in the gene knockdown strains. qRT-PCR data of transcripts in the gene knockdown and control strains are shown. The level of each transcript is expressed as a percentage relative to that of the control strain. Experiments were performed in triplicates; *n* = 3. (B) Growth curves of EhKDHRgs and control strains. Experiments were performed in triplicates; *n* = 2. Download FIG S6, TIF file, 0.8 MB. Copyright © 2021 Mi-ichi et al. 2021 Mi-ichi et al. https://creativecommons.org/licenses/by/4.0/ This content is distributed under the terms of the Creative Commons Attribution 4.0 International license.

Our lipidomic analysis detected PE-Cers, PI-Cers, and SMs, the precursors of which are ceramides (Fig. 2A and Fig. S1B to D), which is consistent with the previous studies (29, 30). Furthermore, a drastic increase of some very-long-chain PE-Cer species, such as PE-Cer 18:0;2O/26:0 and PE-Cer 18:0;2O/28:1, was observed during *E*. *invadens* encystation (Fig. S1B), although the total amount of PE-Cers in cells did not change (Fig. 2A). Because changes in the level of PE-Cer-NDSs and Cer-NDSs levels were well correlated during the course of cyst formation (Fig. 2C andS1A and B), PE-Cer-NDSs appeared to be synthesized *de novo* via Cer-NDSs. Note that previous studies determined the effects of E. histolytica and *E*. *invadens CerS2* gene knockdown or overexpression on trophozoite proliferation, encystation, and excystation (25, 26). The observed phenotypes, at least for E. histolytica trophozoite proliferation, were inconsistent with our present results from the E. histolytica genetic study (Fig. S4B). We attribute this inconsistency to the functional redundancy among EhCerS2, -5, and -6. This genetic redundancy may also affect the encystation and excystation, because *E. invadens* possesses all of these counterparts (AmoebaDB) (26) (Fig. 1B). However, the possibility that CerS2 specifically functions in these processes cannot be ruled out; therefore, alternative approaches, such as pharmacological blockage of specific CerS, are required for elucidating the roles of Cer-NDS species, products of CerS, during *Entamoeba* encystation and excystation. Taken together, *Entamoeba* provides the necessary diversity of sphingolipids, such as Cer, PE-Cer, PI-Cer, and SM. However, the precise physiology of these sphingolipids in *Entamoeba*, including identification and characterization of sphingolipid synthase(s) and the uptake mechanism of SM from the host, needs to be unraveled.

As well as ceramides, sphingolipid and glycerophospholipid diversity are generated by variations in acyl chains, i.e., the number of carbon atoms and the level of unsaturation (Fig. S1E to K). The acyl chain variations in these lipids are principally introduced by a ubiquitous enzyme, acyl-CoA synthetase, which uses various fatty acids as a substrate. Organisms typically utilize fatty acids *per se*, which are either scavenged from the external milieu or synthesized by a *de novo* pathway. After elongation and desaturation by fatty acid elongases and desaturases, respectively, these provide fatty acids. Unlike typical organisms, such as human and yeast, *Entamoeba* relies totally on the external milieu as the fatty acid source because genes for neither type I nor II fatty acid synthases, responsible for *de novo* synthesis, are present in the genome (34, 40, 41). Furthermore, fatty acid desaturases are not encoded. In contrast, all enzymes necessary for fatty acid elongation, which proceeds via a four-step biochemical cycle (42, 43), are encoded in *Entamoeba* genomes (AmoebaDB) (34, 40) (see Fig. S7A). Consistently, during encystation, significant upregulation of *E. invadens* genes that encode enzymes involved in fatty acid elongation was observed (Fig. S7B). Notably, knockdown of the gene encoding the second enzyme of the pathway in E. histolytica produced a severe growth defect. Therefore, *Entamoeba* fatty acid elongation, along with other lipid metabolism, such as sphingolipid and sulfolipid metabolism, is crucial for maintenance of the life cycle (this study, 34).

10.1128/mSphere.00174-21.7 FIG S7 *Entamoeba* fatty acid elongation. (A) Scheme of the *Entamoeba* fatty acid elongation cycle. AmoebaDB gene IDs for E. histolytica and *E. invadens* enzymes are indicated by red and blue colors, respectively. (B) Transcriptional changes of the genes encoding the enzymes involved in fatty acid elongation during *Entamoeba* encystation. Expression levels are shown as fold changes at the indicated time points after the induction of encystation relative to the level at time 0 h. Experiments were performed in triplicates, and representative data are shown from three independent experiments. Download FIG S7, TIF file, 1.8 MB. Copyright © 2021 Mi-ichi et al. 2021 Mi-ichi et al. https://creativecommons.org/licenses/by/4.0/ This content is distributed under the terms of the Creative Commons Attribution 4.0 International license.

In conclusion, we have shown the overall scheme of *Entamoeba* sphingolipid metabolism and its unique features. These findings substantiate the importance of lipid metabolism in *Entamoeba* encystation and indicate a new role for ceramides in organism homeostasis. This contributes not only to the advances in understanding *Entamoeba* physiology but also to the field of sphingolipid and membrane biology.

## MATERIALS AND METHODS

### Parasite cultures.

E. histolytica (G3 and HM-1:IMSS cl6) were routinely maintained as previously described (44). *E. invadens* (IP-1) was routinely maintained in a glass tube filled with 6 ml BI-S-33 (proliferation medium). To induce encystation, 2.5 × 10^5^ *E. invadens* trophozoites were seeded in a Nunc cell culture flask with a solid cap (catalog number [no.] 163371; Thermo Fisher Scientific, Waltham, MA, USA) filled with 56 ml BI-S-33 medium and cultivated at 26°C for 5 days. Trophozoites were harvested from the required numbers of flasks and transferred to encystation medium (37) at a final concentration of 6 × 10^5^/ml.

### LC-MS/MS-based lipidomics.

*E*. *invadens* cyst formation was induced as previously described in either the absence or presence of 1 μM myriocin (37). One micromolar myriocin was freshly diluted from 5 mM stock, which was prepared by dissolving myriocin powder (Cayman, MI, USA) in dimethyl sulfoxide (DMSO) and stored at −30°C. Sample containing 0.02% DMSO was used as a control of myriocin treatment. Briefly, trophozoites suspended in encystation medium (6 × 10^5^ cells/ml) were seeded in 24-well culture plates (2 ml per well) and sealed as described (45) using Parafilm (Bemis Company, Inc., Oshkosh, WI, USA). Then, plates were incubated at 26°C for the period indicated in the text and figures. Cell pellets from two wells of a 24-well plate were collected in a single 15-ml tube using 10 ml phosphate-buffered saline (PBS) and then centrifuged at 770 × *g* for 5 min at 4°C. The cell pellet was washed with 6 ml PBS and resuspended in 4 ml PBS. One milliliter of the cell suspension was then dispensed into each of four 1.5-ml tubes, and cells were repelleted by centrifugation. Cell pellets in tubes were kept at −80°C until use.

For E. histolytica transformants, stably subculturing cells (1.5 × 10^6^) in the presence of 20 μg/ml G418 disulfate (Nacalai Tesque, Kyoto, Japan) were collected in a 5-ml tube by centrifugation at 440 × *g* for 5 min at 4°C. The cell pellet of each transformant was washed with 4 ml PBS and resuspended in 1.5 ml PBS. Five hundred microliters of the cell suspension was dispensed into each of three 1.5-ml tubes, and cells were repelleted by centrifugation at 770 × *g* for 5 min at 4°C. Cell pellets were then kept at −80°C until use.

Lipids were extracted from cells using single-phase extraction as previously described (46) with minor modifications. The cell pellet prepared as described above was mixed with 0.5 ml methanol, sonicated for 2 min, and incubated for 1 h at ambient temperature. After 0.2 ml of the obtained suspension was mixed with 0.1 ml CHCl_3_ in a new glass tube, the sample was incubated for 1 h at ambient temperature. Then, 20 μl water was added to the sample, and the mixture was incubated for 15 min at ambient temperature. After the extract was centrifuged at 2,000 × *g* for 10 min at ambient temperature, the supernatants were collected and dried. The obtained lipids were resuspended in 50 μl methanol (MeOH)-CHCl_3_-H_2_O solution (2:1:0.2 [vol/vol/vol]), and were then kept at 4°C until use.

LC-MS/MS analysis was carried out using a quadrupole time of flight mass spectrometer, TripleTOF 6600 (SCIEX, Framingham, MA, USA) coupled with an ACQUITY ultraperformance liquid chromatography (UPLC) system (Waters, Milford, MA, USA). All analyses were performed using data-dependent MS/MS acquisition (DDA) at the high-resolution mode in MS1 and at the high sensitivity mode in MS2. The UPLC peptide ethylene-bridged hybrid (BEH) C_18_ (50 by 2.1 mm; 1.7 μm) column was maintained at 45°C at a flow rate of 0.3 ml/min. The LC separation was performed with a gradient elution of mobile phase A (methanol-acetonitrile-water, 1:1:3 [vol/vol/vol] containing 5 mM ammonium acetate [Wako Chemicals, Osaka, Japan] and 10 nM EDTA [Dojindo, Kumamoto, Japan]) and mobile phase B (isopropanol containing 5 mM ammonium acetate and 10 nM EDTA). The LC gradient and mass spectrometer settings were the same as previously described (46). The data analysis was performed as previously described (20). The obtained data were, as a result, normalized by adjusting the cell numbers processed; for encysting cells, the cell numbers were those treated for encystation induction, whereas for transformants, those treated for lipid extraction were used.

### Metabolic labeling of *E*. *invadens* and lipid analysis.

*E. invadens* trophozoites suspended in proliferation medium (1.5 × 10^5^/ml) or encystation medium (6 × 10^5^ cells/ml) were seeded in 96-well culture plates (240 μl per well). After adding U-^14^C-labeled l-serine (173.6 mCi/mmol) (Moravek, Brea, CA, USA) to each well (final radioactivity, 3 μCi/ml), the plates were sealed and incubated at 26°C for the period indicated as described above. For each time indicated, cell cultures from four wells of a 96-well plate were collected in a single 6-ml glass tube, and cells were pelleted by centrifugation at 1,500 × *g* for 5 min at 4°C. The cell pellet in each tube was washed twice with PBS. Then lipids were extracted by successive addition of 3.8 ml chloroform-methanol-0.15 N HCl (5:10:4 [vol/vol/vol]), 1 ml chloroform, and 1 ml 1% KCl (wt/vol deionized water) with thorough mixing at each addition. Phases were separated by centrifugation at 770 × *g* for 5 min at ambient temperature, and the organic phase was recovered and dried. The lipids extracted from 2.88 × 10^5^ cells were resolved by thin-layer chromatography (TLC) on Silica Gel 60 high-performance TLC plates (Merck, Darmstadt, Germany) with chloroform-methanol-15 N NH_3_ (60:35:8 [vol/vol/vol]). Each spot on the TLC plates was quantified using a Fuji imaging analyzer and Multi Gauge 2.2 software (FLA-7000; Fujifilm, Tokyo, Japan).

### Alkaline treatment of lipids.

The lipids obtained from 2.88 × 10^5^ cells, as described above, were suspended in 600 μl 0.1 M KOH in chloroform-methanol (2:1 [vol/vol]) and incubated for 2 h at 37°C. After incubation, the lipid solution was sequentially mixed with 21 μl 4 M formic acid, 200 μl chloroform, and 400 μl deionized water. Then, the phases were separated by centrifugation at 770 × *g* for 5 min at ambient temperature, and the organic phase was recovered, dried, and dissolved in 50 μl chloroform-methanol (1:1 [vol/vol]) (47). The obtained lipids were resolved by TLC on Silica Gel 60 high-performance TLC plates (Merck, Darmstadt, Germany) with chloroform-methanol-15 N NH_3_ (60:35:8 [vol/vol/vol]). Each spot on the TLC plates was analyzed as described above.

### Real-time qRT-PCR.

Real-time qRT-PCR was performed as previously described (48) with minor modifications. Total RNA from *Entamoeba* cells was extracted with RNAiso Plus (TaKaRa Bio Inc., Kyoto, Japan), and then cDNA was synthesized using the ReverTra Ace qPCR RT master mix with genomic DNA (gDNA) remover (Toyobo Co. Ltd., Osaka, Japan). Real-time PCR was performed using StepOnePlus (Thermo Fisher Scientific, Waltham, MA, USA), Thunderbird SYBR qPCR mix (Toyobo), and appropriate primer sets (see Table S3 in the supplemental material). For encysting *E. invadens* cell analysis, the cell pellets from one aliquot prepared for lipidomics were suspended in 1 ml RNAiso Plus (TaKaRa), and the resulting samples were similarly processed and analyzed.

10.1128/mSphere.00174-21.10 TABLE S3 Primers used in this study. Download Table S3, PDF file, 0.03 MB. Copyright © 2021 Mi-ichi et al. 2021 Mi-ichi et al. https://creativecommons.org/licenses/by/4.0/ This content is distributed under the terms of the Creative Commons Attribution 4.0 International license.

### Gene knockdown in E. histolytica.

Construction of pSAP2-g-multi-based plasmids for gene silencing using appropriate primers (Table S3) was performed essentially as described previously (34). Plasmid transfection into E. histolytica (G3) trophozoites using Lipofectamine LTX and establishment of stable transformants were performed as described (34). The knockdown levels of the targeted genes in the established transformants were evaluated by real-time qRT-PCR using suitable primer sets (Table S3) as described above.

### Overexpression in E. histolytica.

Overexpression of HA-tagged proteins was achieved using the pEhEx-m-HA vector, which was derived from pEhEx-HA (36). The EcoRI-BglII fragment of pEhEx-HA was replaced with a newly PCR-amplified 5′ conserved sequence (CS) region appended with EcoRI and NheI-HindIII-BglII sites at the either end. PCR amplicons harboring open reading frames (ORFs) of target genes were obtained using suitable primers sets (Table S3), digested with NheI and BglII, and inserted into the corresponding sites of pEhEx-m-HA. The resulting correct plasmids were then introduced into E. histolytica (HM-1:IMSS cl6) trophozoites, and stable transformants were established as described above. The levels of overexpression of the targeted genes in the established transformants were evaluated by real-time qRT-PCR using suitable primer sets (Table S3) as described above.

### Cell growth assay.

The cell growth assay was performed as described previously (48, 49) except that the starting cell number was 1,666 or 3,000 trophozoites/ml for overexpression or gene knockdown transformants, respectively. The IC_50_ of myriocin for trophozoite proliferation was also similarly determined. In detail, trophozoites of either E. histolytica (HM-1:IMSS cl6) or *E. invadens* (IP-1) were collected from routine cultures and suspended in proliferation medium at 3,333 cells/ml. Three milliliters of the cell suspension was dispensed into a 7-ml glass tube, which was filled with 6 ml proliferation medium containing various concentrations of myriocin. Each myriocin stock, prepared as described below, was added to the medium at 1/100 before trophozoite inoculation. For a solvent control, in place of the myriocin stock solution, DMSO was added at final concentration of 1% (vol/vol). After incubating for 72 h, viable cells were manually counted under a phase-contrast microscope.

### Flow cytometry and fluorescence and electron microscopy.

*E*. *invadens* trophozoites treated for encystation were suspended in encystation medium (37) containing either various concentrations of myriocin or the solvent control, DMSO. Note that the DMSO content in all wells was 1% (vol/vol). Then, the cell suspensions were seeded in 96-well culture plates, sealed, and incubated at 26°C for the designated period, as described above. The medium containing myriocin was prepared by adding each myriocin stock solution at 1/100 (vol/vol). The series of stock solutions was prepared by serial dilution with DMSO from a 5 mM stock.

For flow cytometry using Evans blue (EB) and calcofluor (CF), cells in the above-described cultures were treated and processed, and the obtained data were analyzed as described previously (37). The IC_50_ of myriocin for the cyst formation at 72 h after inducing encystation was also determined by this flow cytometry method.

For fluorescence microscopy, a portion of the flow cytometry samples (1 μM myriocin and control) was examined at 24 h under a fluorescence microscope (Zeiss Axio Imager 2; Carl Zeiss, Germany) equipped with a Zeiss AxioCam 305 mono camera (Carl Zeiss). The obtained images were processed using ZEN software (Carl Zeiss).

Transmission electron microscopy analysis, based on a rapid freezing and freeze-fixation method, was outsourced to Tokai Electron Microscopy, Inc. (Nagoya, Japan). The cells treated for encystation were cultivated either in the presence of 1 μM myriocin or DMSO for 24 h in 6 wells of a 96-well plate as described above. Then, the cells were collected in a single 1.5-ml tube using 1 ml PBS and pelleted by centrifugation at 5,200 × *g* for 1 min at 4°C. Each cell pellet was sandwiched between copper disks and quickly frozen in liquid propane at −175°C. The resulting samples were then freeze-substituted with 2% glutaraldehyde and 1% tannic acid in ethanol containing 2% distilled water (vol/vol) at −80°C for 48 h. Subsequently, they were transferred to a −20°C freezer and kept at −20°C for 3 h, followed by warming to 4°C by 4 h. The samples were then dehydrated for 30 min three times in absolute ethanol at ambient temperature and left in absolute ethanol at ambient temperature overnight. On the following day, the samples were soaked for 30 min twice in propylene oxide (PO) and once in a mixture of PO and resin (Quetol-812; Nisshin EM Co., Tokyo, Japan) (70:30 [vol/vol]) for 1 h. The tube cap was left open overnight to completely volatilize the PO. Then, the samples were freshly soaked in 100% resin and incubated at 60°C for 48 h to polymerize the resin. The samples embedded in the polymerized resins were sectioned at an ultrathin thickness of 70 nm using an ultramicrotome (Ultracut UCT; Leica, Vienna, Austria). These samples were stained with 2% uranyl acetate at ambient temperature for 15 min and washed with distilled water. Then, the samples were secondarily stained with lead stain solution (Sigma-Aldrich, St. Louis, MO, USA) at ambient temperature for 3 min. The resulting samples were then examined at 100 kV acceleration voltage under a transmission electron microscope (JEM-1400 Plus; JEOL, Tokyo, Japan). Images were acquired using a charge-coupled-device (CCD) camera (EM-14830RUBY2; JEOL).

### Data availability.

All raw mass spectrometry data are freely available on the RIKEN DROP Met website (http://prime.psc.riken.jp/menta.cgi/prime/drop_index), under index number DM0036.

## References

[1] Haque R, Huston CD, Hughes M, Houpt E, Petri WA, Jr. 2003. Amebiasis. N Engl J Med 348:1565–1573. doi:10.1056/NEJMra022710. 12700377

[2] Lozano R, Naghavi M, Foreman K, Lim S, Shibuya K, Aboyans V, Abraham J, Adair T, Aggarwal R, Ahn SY, Alvarado M, Anderson HR, Anderson LM, Andrews KG, Atkinson C, Baddour LM, Barker-Collo S, Bartels DH, Bell ML, Benjamin EJ, Bennett D, Bhalla K, Bikbov B, Bin Abdulhak A, Birbeck G, Blyth F, Bolliger I, Boufous S, Bucello C, Burch M, Burney P, Carapetis J, Chen H, Chou D, Chugh SS, Coffeng LE, Colan SD, Colquhoun S, Colson KE, Condon J, Connor MD, Cooper LT, Corriere M, Cortinovis M, de Vaccaro KC, Couser W, Cowie BC, Criqui MH, Cross M, Dabhadkar KC, et al. 2012. Global and regional mortality from 235 causes of death for 20 age groups in 1990 and 2010: a systematic analysis for the Global Burden of Disease Study 2010. Lancet 380:2095–2128. doi:10.1016/S0140-6736(12)61728-0. 23245604 PMC10790329

[3] Quach J, St-Pierre J, Chadee K. 2014. The future for vaccine development against Entamoeba histolytica. Hum Vaccin Immunother 10:1514–1521. doi:10.4161/hv.27796. 24504133 PMC5396225

[4] Eichinger D. 2001. Encystation in parasitic protozoa. Curr Opin Microbiol 4:421–426. doi:10.1016/s1369-5274(00)00229-0. 11495805

[5] Mi-Ichi F, Yoshida H, Hamano S. 2016. *Entamoeba* encystation: new targets to prevent the transmission of amebiasis. PLoS Pathog 12:e1005845. doi:10.1371/journal.ppat.1005845. 27764256 PMC5072687

[6] Watanabe K, Petri WA, Jr. 2015. Molecular biology research to benefit patients with *Entamoeba histolytica* infection. Mol Microbiol 98:208–217. doi:10.1111/mmi.13131. 26173474

[7] Barker DC, Deutsch K. 1958. The chromatoid body of *Entamoeba invadens*. Exp Cell Res 15:604–610. doi:10.1016/0014-4827(58)90108-3. 13609637

[8] Barker DC, Svihla G. 1964. Localization of cytoplasmic nucleic acid during growth and encystment of *Entamoeba invadens*. J Cell Biol 20:389–398. doi:10.1083/jcb.20.3.389. 14128044 PMC2106411

[9] Eichinger D. 1997. Encystation of entamoeba parasites. Bioessays 19:633–639. doi:10.1002/bies.950190714. 9230696

[10] Tanyuksel M, Petri WA, Jr. 2003. Laboratory diagnosis of amebiasis. Clin Microbiol Rev 16:713–729. doi:10.1128/cmr.16.4.713-729.2003. 14557296 PMC207118

[11] Mousa EAA, Sakaguchi M, Nakamura R, Abdella OH, Yoshida H, Hamano S, Mi-Ichi F. 2020. The dynamics of ultrastructural changes during *Entamoeba invadens* encystation. Parasitology 147:1305–1312. doi:10.1017/S0031182020001079. 32660674 PMC10317766

[12] Chavez-Munguia B, Martinez-Palomo A. 2011. High-resolution electron microscopical study of cyst walls of *Entamoeba* spp. J Eukaryot Microbiol 58:480–486. doi:10.1111/j.1550-7408.2011.00576.x. 21883633

[13] Chavez-Munguia B, Omana-Molina M, Gonzalez-Lazaro M, Gonzalez-Robles A, Cedillo-Rivera R, Bonilla P, Martinez-Palomo A. 2007. Ultrastructure of cyst differentiation in parasitic protozoa. Parasitol Res 100:1169–1175. doi:10.1007/s00436-006-0447-x. 17252271

[14] Samuelson J, Bushkin GG, Chatterjee A, Robbins PW. 2013. Strategies to discover the structural components of cyst and oocyst walls. Eukaryot Cell 12:1578–1587. doi:10.1128/EC.00213-13. 24096907 PMC3889564

[15] Manna D, Singh U. 2019. Nuclear factor Y (NF-Y) modulates encystation in entamoeba via stage-specific expression of the NF-YB and NF-YC subunits. mBio 10:e00737-19. doi:10.1128/mBio.00737-19. 31213550 PMC6581852

[16] Campos-Gongora E, Ebert F, Willhoeft U, Said-Fernandez S, Tannich E. 2004. Characterization of chitin synthases from *Entamoeba*. Protist 155:323–330. doi:10.1078/1434461041844204. 15552059

[17] Jeelani G, Sato D, Husain A, Escueta-de Cadiz A, Sugimoto M, Soga T, Suematsu M, Nozaki T. 2012. Metabolic profiling of the protozoan parasite *Entamoeba invadens* revealed activation of unpredicted pathway during encystation. PLoS One 7:e37740. doi:10.1371/journal.pone.0037740. 22662204 PMC3360610

[18] Samanta SK, Ghosh SK. 2012. The chitin biosynthesis pathway in *Entamoeba* and the role of glucosamine-6-P isomerase by RNA interference. Mol Biochem Parasitol 186:60–68. doi:10.1016/j.molbiopara.2012.09.011. 23058929

[19] Harayama T, Riezman H. 2018. Understanding the diversity of membrane lipid composition. Nat Rev Mol Cell Biol 19:281–296. doi:10.1038/nrm.2017.138. 29410529

[20] Tsugawa H, Ikeda K, Takahashi M, Satoh A, Mori Y, Uchino H, Okahashi N, Yamada Y, Tada I, Bonini P, Higashi Y, Okazaki Y, Zhou Z, Zhu ZJ, Koelmel J, Cajka T, Fiehn O, Saito K, Arita M, Arita M. 2020. A lipidome atlas in MS-DIAL 4. Nat Biotechnol 38:1159–1163. doi:10.1038/s41587-020-0531-2. 32541957

[21] Hannun YA, Obeid LM. 2018. Sphingolipids and their metabolism in physiology and disease. Nat Rev Mol Cell Biol 19:175–191. doi:10.1038/nrm.2017.107. 29165427 PMC5902181

[22] Kihara A. 2016. Synthesis and degradation pathways, functions, and pathology of ceramides and epidermal acylceramides. Prog Lipid Res 63:50–69. doi:10.1016/j.plipres.2016.04.001. 27107674

[23] Vargas-Villarreal J, Palacios-Corona R, Hernández-Luna C, Mata-Cárdenas BD, Torres de la Cruz VM, Cortés-Gutiérrez EI, González-Salazar F, Garza-González JN, Escobedo-Guajardo BL, Said-Fernández S. 2010. *Entamoeba histolytica*: soluble and membrane-associated neutral sphingomyelinase-C and other unidentified esterase activity. Exp Parasitol 125:394–399. doi:10.1016/j.exppara.2010.03.010. 20350542

[24] Jin J, Hou Q, Mullen TD, Zeidan YH, Bielawski J, Kraveka JM, Bielawska A, Obeid LM, Hannun YA, Hsu YT. 2008. Ceramide generated by sphingomyelin hydrolysis and the salvage pathway is involved in hypoxia/reoxygenation-induced Bax redistribution to mitochondria in NT-2 cells. J Biol Chem 283:26509–26517. doi:10.1074/jbc.M801597200. 18676372 PMC2546549

[25] Jauregui-Wade JM, Cerbon-Solorzano J, Avila-Garcia R, Ayala-Sumuano JT, Valdes J. 2020. Ceramide synthase 2 knockdown suppresses trophozoite growth, migration, *in vitro* encystment and excystment of *Entamoeba invadens*. Biochem Biophys Res Commun 524:135–141. doi:10.1016/j.bbrc.2020.01.093. 31980165

[26] Avila-Garcia R, Valdes J, Jauregui-Wade JM, Ayala-Sumuano JT, Cerbon-Solorzano J. 2020. The metabolic pathway of sphingolipids biosynthesis and signaling in *Entamoeba histolytica*. Biochem Biophys Res Commun 522:574–579. doi:10.1016/j.bbrc.2019.11.116. 31785811

[27] Jauregui-Wade JM, Valdes J, Ayala-Sumuano JT, Avila-Garcia R, Cerbon-Solorzano J. 2019. *De novo* synthesis of sphingolipids plays an important role during *in vitro* encystment of *Entamoeba invadens*. Biochem Biophys Res Commun 508:1031–1037. doi:10.1016/j.bbrc.2018.12.005. 30545628

[28] Cerbon J, Olguin T, Alvarez-Grave PR, Lopez-Sanchez RC. 2009. *Entamoeba invadens*: sphingolipids metabolic regulation is the main component of a PKC signaling pathway in controlling cell growth and proliferation. Exp Parasitol 122:106–111. doi:10.1016/j.exppara.2009.02.011. 19249300

[29] Aley SB, Scott WA, Cohn ZA. 1980. Plasma membrane of *Entamoeba histolytica*. J Exp Med 152:391–404. doi:10.1084/jem.152.2.391. 6249883 PMC2185943

[30] van Vliet HH, den Kamp JA, van Deenen LL. 1975. Phospholipids of *Entamoeba invadens*. Arch Biochem Biophys 171:55–64. doi:10.1016/0003-9861(75)90006-5. 1190798

[31] Mizutani Y, Mitsutake S, Tsuji K, Kihara A, Igarashi Y. 2009. Ceramide biosynthesis in keratinocyte and its role in skin function. Biochimie 91:784–790. doi:10.1016/j.biochi.2009.04.001. 19364519

[32] Chatterjee A, Ghosh SK, Jang K, Bullitt E, Moore L, Robbins PW, Samuelson J. 2009. Evidence for a "wattle and daub" model of the cyst wall of entamoeba. PLoS Pathog 5:e1000498. doi:10.1371/journal.ppat.1000498. 19578434 PMC2698119

[33] Sanchez L, Enea V, Eichinger D. 1994. Identification of a developmentally regulated transcript expressed during encystation of *Entamoeba invadens*. Mol Biochem Parasitol 67:125–135. doi:10.1016/0166-6851(94)90102-3. 7838173

[34] Mi-Ichi F, Miyamoto T, Yoshida H. 2017. Uniqueness of *Entamoeba* sulfur metabolism: sulfolipid metabolism that plays pleiotropic roles in the parasitic life cycle. Mol Microbiol 106:479–491. doi:10.1111/mmi.13827. 28884488

[35] Bracha R, Nuchamowitz Y, Anbar M, Mirelman D. 2006. Transcriptional silencing of multiple genes in trophozoites of *Entamoeba histolytica*. PLoS Pathog 2:e48. doi:10.1371/journal.ppat.0020048. 16733544 PMC1464398

[36] Saito-Nakano Y, Yasuda T, Nakada-Tsukui K, Leippe M, Nozaki T. 2004. Rab5-associated vacuoles play a unique role in phagocytosis of the enteric protozoan parasite *Entamoeba histolytica*. J Biol Chem 279:49497–49507. doi:10.1074/jbc.M403987200. 15347665

[37] Mi-Ichi F, Miyake Y, Tam VK, Yoshida H. 2018. A flow cytometry method for dissecting the cell differentiation process of entamoeba encystation. Front Cell Infect Microbiol 8:250. doi:10.3389/fcimb.2018.00250. 30087858 PMC6066566

[38] Arroyo-Begovich A, Carabez-Trejo A, Ruiz-Herrera J. 1980. Identification of the structural component in the cyst wall of *Entamoeba invadens*. J Parasitol 66:735–741. doi:10.2307/3280662. 7463242

[39] Baba M. 2008. Electron microscopy in yeast. Methods Enzymol 451:133–149. doi:10.1016/S0076-6879(08)03210-2. 19185718

[40] Castellanos-Castro S, Bolanos J, Orozco E. 2020. Lipids in *Entamoeba histolytica*: host-dependence and virulence factors. Front Cell Infect Microbiol 10:75. doi:10.3389/fcimb.2020.00075. 32211340 PMC7075943

[41] Clark CG, Alsmark UC, Tazreiter M, Saito-Nakano Y, Ali V, Marion S, Weber C, Mukherjee C, Bruchhaus I, Tannich E, Leippe M, Sicheritz-Ponten T, Foster PG, Samuelson J, Noel CJ, Hirt RP, Embley TM, Gilchrist CA, Mann BJ, Singh U, Ackers JP, Bhattacharya S, Bhattacharya A, Lohia A, Guillen N, Duchene M, Nozaki T, Hall N. 2007. Structure and content of the *Entamoeba histolytica* genome. Adv Parasitol 65:51–190. doi:10.1016/S0065-308X(07)65002-7. 18063096

[42] Sassa T, Kihara A. 2014. Metabolism of very long-chain fatty acids: genes and pathophysiology. Biomol Ther (Seoul) 22:83–92. doi:10.4062/biomolther.2014.017. 24753812 PMC3975470

[43] Nugteren DH. 1965. The enzymic chain elongation of fatty acids by rat-liver microsomes. Biochim Biophys Acta 106:280–290. doi:10.1016/0005-2760(65)90036-6. 4379659

[44] Mi-Ichi F, Abu Yousuf M, Nakada-Tsukui K, Nozaki T. 2009. Mitosomes in *Entamoeba histolytica* contain a sulfate activation pathway. Proc Natl Acad Sci U S A 106:21731–21736. doi:10.1073/pnas.0907106106. 19995967 PMC2799805

[45] Suresh S, Ehrenkaufer G, Zhang H, Singh U. 2016. Development of RNA interference trigger-mediated gene silencing in *Entamoeba invadens*. Infect Immun 84:964–975. doi:10.1128/IAI.01161-15. 26787723 PMC4807475

[46] Tsugawa H, Ikeda K, Tanaka W, Senoo Y, Arita M, Arita M. 2017. Comprehensive identification of sphingolipid species by *in silico* retention time and tandem mass spectral library. J Cheminform 9:19. doi:10.1186/s13321-017-0205-3. 28316657 PMC5352698

[47] Ohno Y, Nakamichi S, Ohkuni A, Kamiyama N, Naoe A, Tsujimura H, Yokose U, Sugiura K, Ishikawa J, Akiyama M, Kihara A. 2015. Essential role of the cytochrome P450 CYP4F22 in the production of acylceramide, the key lipid for skin permeability barrier formation. Proc Natl Acad Sci U S A 112:7707–7712. doi:10.1073/pnas.1503491112. 26056268 PMC4485105

[48] Mi-Ichi F, Miyamoto T, Takao S, Jeelani G, Hashimoto T, Hara H, Nozaki T, Yoshida H. 2015. Entamoeba mitosomes play an important role in encystation by association with cholesteryl sulfate synthesis. Proc Natl Acad Sci U S A 112:E2884–E2890. doi:10.1073/pnas.1423718112. 25986376 PMC4460517

[49] Mi-Ichi F, Makiuchi T, Furukawa A, Sato D, Nozaki T. 2011. Sulfate activation in mitosomes plays an important role in the proliferation of *Entamoeba histolytica*. PLoS Negl Trop Dis 5:e1263. doi:10.1371/journal.pntd.0001263. 21829746 PMC3149026

