# Supplementary material for: Stage-Specific De Novo Synthesis of Very-Long-Chain Dihydroceramides Confers Dormancy to Entamoeba Parasites
Source: mSphere. 2021 Mar 17;6(2):e00174-21. doi: 10.1128/mSphere.00174-21 (PMC8546694; doi:10.1128/mSphere.00174-21)
Supplement: TABLE S1 [file msphere.00174-21-st001.pdf]

Table. S1

Ceramide Species	Sample 1		Sample 2		Sample 3	
	Ranking	%	Ranking	%	Ranking	%
Cer 18:0;2O/24:1	1	27.2	1	26.1	1	30.9
Cer 18:0;2O/26:0	2	10.3	3	7.8	3	8.3
Cer 18:0;2O/24:0	3	7.5	2	9.3	2	9.6
Cer 16:0;2O/30:2	4	6.6	6	5.1	4	6.5
Cer 19:0;2O/24:1	5	5.9	10	3.7	7	4.1
Cer 18:0;2O/28:1	6	4.7	4	7.8	5	4.9
Cer 18:0;2O/30:2	7	3.9	5	6.1	9	3.7
Cer 18:0;2O/28:0	8	3.7	11	3.5	10	3.0
Cer 16:0;2O/28:1	9	3.6	7	4.7	8	3.9
Cer 18:0;2O/16:0	10	3.4	8	4.4	6	4.1
Cer 18:0;2O/30:1	11	2.1	9	4.1	15	1.4
Cer 19:0;2O/28:0	12	2.1	13	1.6	12	1.8
Cer 17:0;2O/24:1	13	1.8	12	1.7	11	2.1
Cer 19:0;2O/30:2	14	1.8	14	1.4	14	1.5
Cer 19:0;2O/26:0	15	1.7	15	1.3	13	1.8
others		13.7		11.5		12.5
