# Supplementary material for: Stage-Specific De Novo Synthesis of Very-Long-Chain Dihydroceramides Confers Dormancy to Entamoeba Parasites
Source: mSphere. 2021 Mar 17;6(2):e00174-21. doi: 10.1128/mSphere.00174-21 (PMC8546694; doi:10.1128/mSphere.00174-21)
Supplement: TABLE S3 [file msphere.00174-21-st003.pdf]

Table. S3

Purpose	Target gene	Name	Sequence	Direction	Restriction site indicated by underline
qRT-PCR (*E. invadens*)	EiSPT3A	QT-EiSPT3A-F	5'-TTGTGGGAACATGGAAATGG-3'	sense	
		QT-EiSPT3A-R	5'-CAGCACGGCATGTAAATGGT-3'	antisense	
	EiSPT3B	QT-EiSPT3B-F	5'-TCGGTTGTTCCAGTGTACGG-3'	sense	
		QT-EiSPT3B-R	5'-TGCAACCCAATTGAATCTCG-3'	antisense	
	EiKDHR	QT-EiKDHR-F	5'-TCGGCAACCTCAGAATGATG-3'	sense	
		QT-EiKDHR-R	5'-TCCAAGACACGACCCAACAC-3'	antisense	
	EiCerS1	QT-EiCerS1-F	5'-ACTCCACGACATGCCAAATG-3'	sense	
		QT-EiCerS1-R	5'-TCCACCGAGGGTAACCAATC-3'	antisense	
	EiCerS2	QT-EiCerS2-F	5'-TCGAAATGGGCTATCGACCT-3'	sense	
		QT-EiCerS2-R	5'-CCCAGCCGGGATTATGTAGA-3'	antisense	
	EiCerS3	QT-EiCerS3-F	5'-TCATCCCAGCTGCAATCAAC-3'	sense	
		QT-EiCerS3-R	5'-CAGCCCGACAAGTAGCCAAT-3'	antisense	
	EiCerS4	QT-EiCerS4-F	5'-GTATGGGAAAGGCGCTCAAA-3'	sense	
		QT-EiCerS4-R	5'-GGTATCCCAATTCCCAGCAA-3'	antisense	
	EiCerS5	QT-EiCerS5-F	5'-ATGACACGTTGCTCCACCAT-3'	sense	
		QT-EiCerS5-R	5'-GAAGTACATCACGGCGTCCA-3'	antisense	
	EiCerS6	QT-EiCerS6-F	5'-TACGTCGTCGTAACGGCATT-3'	sense	
		QT-EiCerS6-R	5'-GCACGATGCGTCTTTTGGTA-3'	antisense	
	EiFAE1	QT-EiFAE1-F	5'-CCATCGATCAGTGCCATGTT-3'	sense	
		QT-EiFAE1-R	5'-GGAGACCAATCCCGCACTAC-3'	antisense	
	EiFAE2	QT-EiFAE2-F	5'-CCGCGCGTTATCTCATACAA-3'	sense	
		QT-EiFAE2-R	5'-CCACCTGCGTGGATACAGAA-3'	antisense	
	EiFAE3	QT-EiFAE3-F	5'-GAGAAGAGGCGATGCTCGTT-3'	sense	
		QT-EiFAE3-R	5'-ATCATCGCAGACATGCTTGG-3'	antisense	
	EiFAE4	QT-EiFAE4-F	5'-TGCTGGGTTGATCTCATTGG-3'	sense	
		QT-EiFAE4-R	5'-CTGACCTGTCCACCGTACCA-3'	antisense	
	EiKCRA	QT-EiKCRA-F	5'-GGCCTTCGTGAGACAATTCC-3'	sense	
		QT-EiKCRA-R	5'-TCAGTTGCGACGTACCAAGG-3'	antisense	
	EiKCRB	QT-EiKCRB-F	5'-TTTCCCCATGGTTCATTTCA-3'	sense	
		QT-EiKCRB-R	5'-GAACCAATGTGGGCAACAGA-3'	antisense	
	EiHACD	QT-EiHACD-F	5'-AATGGGTGTGTCTGGGGAAG-3'	sense	
		QT-EiHACD-R	5'-AAATGAGGGCCCAGATGATG-3'	antisense	
	EiTECR	QT-EiTECR-F	5'-CAGAACACCAGGCTCACAGG-3'	sense	
		QT-EiTECR-R	5'-AAGCAACACCCATCAAAGGAA-3'	antisense	
	EiRNApol	QT-EiRNApol-F	5'-GTTCTGTCCGCAATGTCAGC-3'	sense	
		QT-EiRNApol-R	5'-TTGGGGTCCTTCCATTTTTC-3'	antisense	
qRT-PCR (*E. histolytica*)	EhSPT1	QT-EhSPT1-F	5'-TGGTGGTTTTGTCGTTGGAC-3'	sense	
		QT-EhSPT1-R	5'-GAGGGGCTGAAGCAGAGAAA-3'	antisense	
	EhSPT2	QT-EhSPT2-F	5'-GCTTGTATTGCGCTTGATGC-3'	sense	
		QT-EhSPT2-R	5'-ATGGGGTGGAATCATTTCCT-3'	antisense	
	EhKDHR	QT-EhKDHR-F	5'-TGGACACTCCAGGTTTTGCT-3'	sense	
		QT-EhKDHR-R	5'-CAAAGTAAAGCATGTGCTGCAT-3'	antisense	
	EhCerS2	QT-EhCerS2-F	5'-TGGGCAATTGACCTTGAAAA-3'	sense	
		QT-EhCerS2-R	5'-GGCTATCAAACCTGCTCGAA-3'	antisense	
	EhCerS3	QT-EhCerS3-F	5'-TGACATGCCAAATCAAGTTCC-3'	sense	
		QT-EhCerS3-R	5'-GAACCAGGAATTCCATCAGGA-3'	antisense	
	EhCerS4	QT-EhCerS4-F	5'-TCTTCCAGGTGGATTTCTTGTG-3'	sense	
		QT-EhCerS4-R	5'-TGAGGGTCTGCAATTCTTCC-3'	antisense	
	EhCerS5	QT-EhCerS5-F	5'-TGGGGTCTTCTTGTTGGAATG-3'	sense	
		QT-EhCerS5-R	5'-CCCAGCATGAGGATCAACAA-3'	antisense	
	EhCerS6	QT-EhCerS6-F	5'-TCCAGAAACTGCTACTTGTGGTT-3'	sense	
		QT-EhCerS6-R	5'-GCATGTGGATCAACAATACCTTG-3'	antisense	
	EhRNApol	QT-EhRNApol-F	5'-TGAAGACCCAACAAGTTTCTGTG-3'	sense	
		QT-EhRNApol-R	5'-GCTTTGTTGTTTTAGGATATGTTGG-3'	antisense	
Gene knockdown in *E. histolytica*	EhSPT1	GS-EhSPT1-F	5'-AACAGGCCTATGGAAAACAACGATAATTATC-3'	sense	*Stu* I
		GS-EhSPT1-R	5'-AATCCGCGGGTTTTTAATGTTTGAATAATTTC-3'	antisense	*Sac* II
	EhSPT2	GS-EhSPT2-F	5'-AATCCGCGGATGGAAAATAACGACAATTATC-3'	sense	*Sac* II
		GS-EhSPT2-R	5'-AAGACTAGTTTTTTAAACAATCAGCAACTTC-3'	antisense	*Spe* I
	EhKDHR	GS-EhKDHR-F	5'-AACAGGCCTATGGGATTTTTCGATTTCC-3'	sense	*Stu* I
		GS-EhKDHR-R	5'-AATCCGCGGCTTCAATAAACCCTGGATG-3'	antisense	*Sac* II
	EhCerS2	GS-EhCerS2-F	5'-AATCCGCGGATGACCCAACGTCCAAATG-3'	sense	*Sac* II
		GS-EhCerS2-R	5'-AAGACTAGTTTGTTTTCCATTACCAAACAAC-3'	antisense	*Spe* I
	EhCerS3	GS-EhCerS3-F	5'-AAGACTAGTATGAAATTCTTTAGTGGATTAC-3'	sense	*Spe* I
		GS-EhCerS3-R	5'-AATGAGCTCCATAATAACTCAAGGTCTTG-3'	antisense	*Sac* I
	EhCerS4	GS-EhCerS4-F	5'-AACAGGCCTATGAAAATGTCTGATATTTCATTTG-3'	sense	*Stu* I
		GS-EhCerS4-R	5'-AATCCGCGGCCACATATATTCTAAATCTTG-3'	antisense	*Sac* II
	EhCerS5	GS-EhCerS5-F	5'-AACAGGCCTATGCAAATCATGAAACAACC-3'	sense	*Stu* I
		GS-EhCerS5-R	5'-AATCCGCGGTTTCCTTGACCAAATAAAGC-3'	antisense	*Sac* II
	EhCerS6	GS-EhCerS6-F	5'-AATCCGCGGATGCCTAAATTAAATGGAGTATG-3'	sense	*Sac* II
		GS-EhCerS6-R	5'-AAGACTAGTTTGTGAAATAGGGAAATCTTC-3'	antisense	*Spe* I
Production of pEhEx-mHA	-	EhEx-vector-1	5'-TACGAATTCATAGATAATTAATTACTTTT-3'		
	-	EhEx-vector-2	5'-TATAGATCTAAGCTTGCTAGCGTTAATGTGTTTGTCAGTTCATTG-3'		
Gene overexpression in *E. histolytica*	EhSPT1	P-EhSPT1-F	5'-AATGCTAGCATGCAAATCATGAAACAACC-3'	sense	*Nhe* I
		P-EhSPT1-R	5'-AATAGATCTTAATTCATTCTTTTGTTTCCC-3'	antisense	*Bgl* II
	EhSPT2	P-EhSPT2-F	5'-AATGCTAGCATGCCTAAATTAAATGGAGTATG-3'	sense	*Nhe* I
		P-EhSPT2-R	5'-AATAGATCTATTTTCTTGTCCAACAGCC-3'	antisense	*Bgl* II
	EhKDHR	P-EhKDHR-F	5'-AATGCTAGCATGAAATTCTTTAGTGGATTACC-3'	sense	*Nhe* I
		P-EhKDHR-R	5'-AATAGATCTTCCTAAAGCATGAGGATCT-3'	antisense	*Bgl* II
	EhCerS2	P-EhCerS2-F	5'-AATGCTAGCATGGAAAATAACGACAATTATC-3'	sense	*Nhe* I
		P-EhCerS2-R	5'-TTAGGATCCATAATTGGAAAGAATATTAATAAATT-3'	antisense	*Bgl* II
	EhCerS3	P-EhCerS3-F	5'-AATGCTAGCATGGAAGATGAAATACTCAATG-3'	sense	*Nhe* I
		P-EhCerS3-R	5'-AATAGATCTAATAAGTTGTTGAGATTTAATAGAG-3'	antisense	*Bgl* II
	EhCerS4	P-EhCerS4-F	5'-AATGCTAGCATGGAAAACAACGATAATTATCAAC-3'	sense	*Nhe* I
		P-EhCerS4-R	5'-TTAGGATCCATTTAATTTAGCAAAAGCTGTAATG-3'	antisense	*Bgl* II
	EhCerS5	P-EhCerS5-F	5'-AATGCTAGCATGAAAATGTCTGATATTTCATTTG-3'	sense	*Nhe* I
		P-EhCerS5-R	5'-TTAGGATCCGTTACCAATAGTATGAGGG-3'	antisense	*Bgl* II
	EhCerS6	P-EhCerS6-F	5'-AATGCTAGCATGACCCAACGTCCAAATG-3'	sense	*Nhe* I
		P-EhCerS6-R	5'-AATAGATCTTTCTACAATAGAATGAATGTCTC-3'	antisense	*Bgl* II
